# Effects of Fentanyl-Laced Cocaine on Circulating Ghrelin, Insulin, and Glucose Levels in Rats

**DOI:** 10.3390/ijms26052341

**Published:** 2025-03-06

**Authors:** Huimei Wei, Elise C. Maul, Annet Kyomuhangi, Shawn Park, Maddilynn L. Mutchler, Chang-Guo Zhan, Fang Zheng

**Affiliations:** 1 Molecular Modeling and Biopharmaceutical Center, College of Pharmacy, University of Kentucky, 789 South Limestone Street, Lexington, KY 40536, USA; 2 Department of Pharmaceutical Sciences, College of Pharmacy, University of Kentucky, 789 South Limestone Street, Lexington, KY 40536, USA

**Keywords:** opioid, ghrelin, glucose, fentanyl, insulin, polydrug use

## Abstract

Opioid mixed with cocaine has been increasingly implicated in overdose deaths, including both intentional co-use of opioid and cocaine and fentanyl-adulterated drug supply. As ghrelin plays an important role in drug reward and can also influence insulin, this study aimed to assess responses of the circulating ghrelin, insulin, and glucose levels to the combined use of fentanyl and cocaine (a polydrug) in rats by performing combined animal behavioral experiments and biochemical analysis. The experimental data consistently revealed that the fentanyl and cocaine co-use can significantly elevate both the acyl-ghrelin and desacyl-ghrelin levels and significantly decrease the insulin level without significant effects on the glucose level. These findings suggest that, like cocaine itself, the fentanyl–cocaine polydrug can self-promote its rewarding effects via elevating the ghrelin level, and that the ghrelin system might serve as a potential pharmacological target for treatment of substance use disorders caused by polysubstance use involving fentanyl and cocaine. Additionally, based on the insulin data obtained in this study, the insulin level was always downregulated significantly and considerably, implying that the fentanyl and cocaine polydrug might have a stronger cardiovascular toxicity to the patients with insulin resistance and diabetes. Further studies are required to examine this possibility.

## 1. Introduction

According to the Centers for Disease Control and Prevention (CDC), drug overdose deaths involving stimulants, including cocaine and methamphetamine (METH), etc., increased from 12,122 in 2015 to 57,497 in 2022 [1]. In 2023, a total of 105,007 drug overdose deaths occurred in the United States [1]. Mixing an opioid with a stimulant is commonly known as speed-balling, and a speedball often has stronger effects than either drug alone. Hence, speed-balling greatly increases the risk of fatal overdose. Overall, speed-balling can be extremely dangerous and more difficult to treat than a single substance abuse [2,3], because speedballs produce a greater rewarding effect than the opioid or stimulant alone [4]. Particularly, the concurrent use of opioids and cocaine has increased in overdose deaths, including both intentional co-use of opioids and cocaine and the use of the fentanyl-adulterated drug supply [5]. Due to fentanyl-adulterated cocaine supply, some cocaine users inadvertently use fentanyl. For the intentional use alone, it was reported recently that half of daily cocaine users knowingly used opioids, and a quarter of daily opioid users knowingly used cocaine [5]. As a result, the mortality among opioid–cocaine co-users was higher than that among the opioid-only users [6]. According to recent reports, 78.6% of drug overdose deaths in 2021 involving cocaine also involved an opioid [7], and nearly 70% of stimulant-involved overdose deaths in 2022 also involved fentanyl [1].

As is well known, cocaine and fentanyl interact with different protein targets (e.g., dopamine transporter for cocaine and opioid receptors for fentanyl) in the brain to produce their drug-rewarding effects. Overall, whereas cocaine as a stimulant stimulates the central nervous system (CNS), fentanyl produces insensibility or a depressant effect on the CNS. Some effects of the two drugs can conceal one another. On the other hand, the drug’s rewarding effects are commonly enhanced by the “hunger hormone” ghrelin, which agonizes the ghrelin (or growth hormone secretagogue) receptor (GHSR) or GHS-R1A [8,9,10,11,12,13]. Notably, GHS-R1A forms hetero-dimers with other receptors, including dopamine receptors (DR)1 and 2 [14,15,16], to enhance the dopamine signaling. Ghrelin is a 28-amino-acid peptide (GSSFLSPEHQKAQQRKESKKPPAKLQPR) that is acylated (*n*-octanoylation) at the Ser3 side chain. With Ser3 *n*-octanoylated, the peptide is acyl-ghrelin (AG)—the commonly known ghrelin. Without *n*-octanoylation, the peptide is known as desacyl-ghrelin (DAG) [17].

The role of ghrelin and its receptor GHS-R1A in drug reward is supported by extensive experimental evidence in the literature. For example, ghrelin-deficient mice showed reduced locomotor responses to cocaine [18]. Administration of a GHS-R1A antagonist (e.g., JMV2959) significantly and dose-dependently attenuated morphine-induced conditioned place preference (CPP) and dopamine sensitization, attenuated METH self-administration, tendency to relapse, and METH-induced CPP [8,9,10,11,12,13], reduced the manifestation of fentanyl CPP, and reduced the fentanyl-seeking/relapse-like behavior in rats on the 12th day of the forced abstinence period [19]. Administration of ghrelin was potentiated, and the GHS-R1A antagonist JMV2959 suppressed cocaine CPP [20], and JMV2959 was efficacious to suppress both cue-reinforced cocaine and oxycodone drug-seeking [21].

Further, according to a recent study [22], circulating ghrelin levels are upregulated by cocaine, and that the ghrelin upregulation plays a critical role in the maintenance of cocaine self-administration and cocaine-seeking. In general, upregulation of ghrelin enhances drug craving, and lever presses for cocaine-associated cues positively correlate with ghrelin levels in rats trained in cocaine self-administration. In comparison, the possible impact of opioids on ghrelin levels is relatively minor. According to a clinical study [23] including 28 patients diagnosed with opioid use disorder (OUD) and 28 normal healthy subjects (control group), there were no significant differences between the two groups in plasma levels of acyl-ghrelin and desacyl-ghrelin, demonstrating that opioids do not upregulate ghrelin in humans.

The objective of the present study was to determine how the combined use of fentanyl and cocaine (a polydrug) will affect circulating ghrelin, insulin, and glucose levels in rats, as these effects may lead to new insights that are valuable for the development of new therapeutic strategies for the treatment of substance use disorders (SUDs) caused by polysubstance use involving fentanyl and cocaine.

## 2. Results

### 2.1. Dose–Response Curve for Rat Self-Administration of Fentanyl in Combination with Cocaine

**Figure 1:**
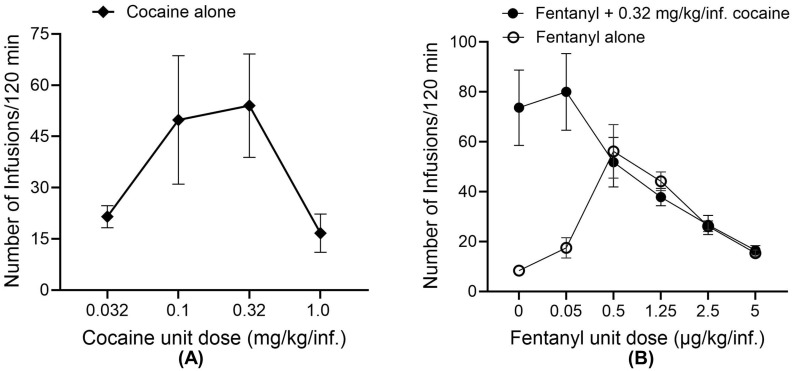
Dose–response curves under the FR1 schedule of reinforcement (n = 6 rats/group) in two-hour sessions of IVSA. (**A**) Number of infusions vs. unit dose of cocaine. (**B**) Number of infusions vs. unit dose of fentanyl with or without 0.32 mg/kg/infusion of cocaine.

This study was designed to assess the effects of the combined use of fentanyl and cocaine in catheterized rats on the circulating levels of ghrelin (including both acyl-ghrelin and desacyl-ghrelin), insulin, and glucose. To select an appropriate dose condition for this study, we first tested various unit dose levels through an intravenous self-administration (IVSA) study in rats using cocaine alone, fentanyl alone, and fentanyl in the presence of cocaine under a fixed ratio-1 (FR1) schedule of reinforcement in two-hour sessions. As shown in Figure 1A, four different unit dose levels (0.032, 0.1, 0.32, and 1 mg/kg/infusion) of cocaine were tested, showing that the cocaine unit dose associated with the maximum number of infusions for IVSA under FR1 was 0.32 mg/kg/infusion, although there was no significant difference between 0.32 and 0.1 mg/kg/infusion in the number of infusions. Hence, we further examined the dose responses of rats to fentanyl in combination with 0.32 mg/kg/infusion of cocaine.

As seen in Figure 1B, six different unit dose levels (0, 0.05, 0.5, 1.25, 2.5, and 5 µg/kg/infusion) of fentanyl were tested for the fentanyl dose response curve alone and then its combination with 0.32 mg/kg/infusion of cocaine. With fentanyl alone, the maximum number of infusions is associated with a unit dose of 0.5 µg/kg/infusion. Hence, we elected to use a fentanyl unit dose (2.5 or 1.25 µg/kg/infusion; see below) higher than 0.5 µg/kg/infusion in its combination with 0.32 mg/kg/infusion of cocaine to ensure that the fentanyl effects are important compared to the cocaine effects. Notably, the presence of 0.32 mg/kg/infusion of cocaine considerably changed the fentanyl dose–response curve. Under the low unit dose (0.05 µg/kg/infusion) of fentanyl, the presence of 0.32 mg/kg/infusion of cocaine considerably increased the number of infusions. Under the higher unit dose (≥0.5 µg/kg/infusion) of fentanyl, the presence of 0.32 mg/kg/infusion of cocaine did not significantly change the number of infusions, suggesting that the fentanyl effect was dominant when its unit dose ≥ 0.5 µg/kg/infusion. Based on the data, an appropriate choice of fentanyl unit dose (2.5 or 1.25 µg/kg/infusion; see below) was used in combination with 0.32 mg/kg/infusion of cocaine.

### 2.2. Fentanyl and Cocaine Co-Use Induced Acute Effects on Ghrelin, Insulin, and Glucose Levels in Rats

To avoid unexpected experiment termination due to animal death, the acute effects of the fentanyl and cocaine co-use were examined by manually administering 25 infusions of the fentanyl–cocaine mixture corresponding to a unit dose of 2.5 µg/kg/infusion of fentanyl and 0.32 mg/kg/infusion of cocaine (test group with 6 rats) or saline (control group with 9 rats) per 2 h (~4.8 min per infusion) and 75 infusions within 6 h to unfasted catheterized rats (through IV catheters that are the same as the ones used for the IVSA experiment discussed above). For each group, the experiment started at 9 a.m. in the morning. Blood samples were collected at four different time points, including 0 (pre-dose), 2 h, 4 h, and 6 h, and analyzed for their blood concentrations of acyl-ghrelin, desacyl-ghrelin, insulin, and glucose. The rats had no access to food or water during the entire session of six hours. With both acyl-ghrelin and desacyl-ghrelin concentrations measured, the total ghrelin concentration was also evaluated as the sum of both. Symptoms of opioid polydrug intoxication, such as sedation, itching, and body wounds caused by biting, were observed at the 4 h time point and later, implying that the accumulated polydrug dose was high at the 4 and 6 h time points. The accumulated doses at various time points are summarized in Table 1.

**Figure 2:**
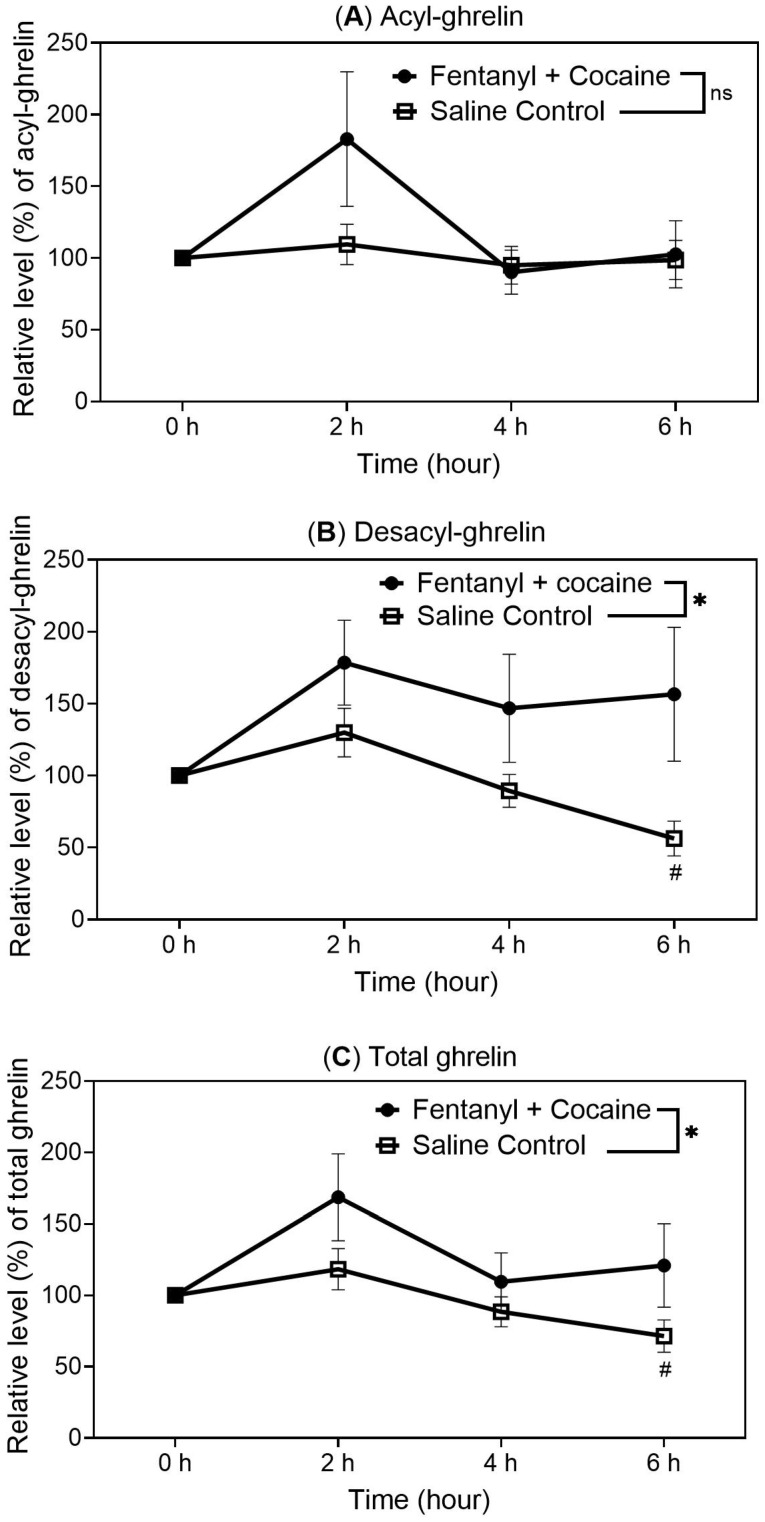
Blood levels of acyl-ghrelin (**A**), desacyl-ghrelin (**B**), and total ghrelin (**C**) in catheterized rats after receiving 25 manual infusions of the fentanyl–cocaine mixture at a unit dose of 2.5 µg/kg/infusion of fentanyl and 0.32 mg/kg/infusion of cocaine (n = 6) or saline (n = 9) per 2 h via IV catheters. The measured average concentrations of acyl-ghrelin, desacyl-ghrelin, and total ghrelin at 0 h (pre-dose) were 238, 302, and 540 pg/mL, respectively. Statistical significance: one-way ANOVA with Dunnett post hoc analysis for comparison of the levels at various time points with the reference level at 0 h—# *p* < 0.05; paired *t*-test for the overall difference between the two groups of rats—* *p* < 0.05 (ns means that the difference is not significant with *p* > 0.05).

As shown in Figure 2, the polydrug (fentanyl and cocaine combination) significantly elevated the desacyl-ghrelin and total ghrelin levels (Figure 2B,C) compared to the saline control group. The acyl-ghrelin level was elevated non-significantly at the 2 h time point according to the *t*-test (Figure 2A), and it returned to the control level at the 4 h and 6 h time points while the symptoms (such as sedation, itching, and body wounds) of opioid polydrug intoxication were observed. This suggests that the opioid polydrug intoxication might have suppressed the potential elevation of acyl-ghrelin level.

**Figure 3:**
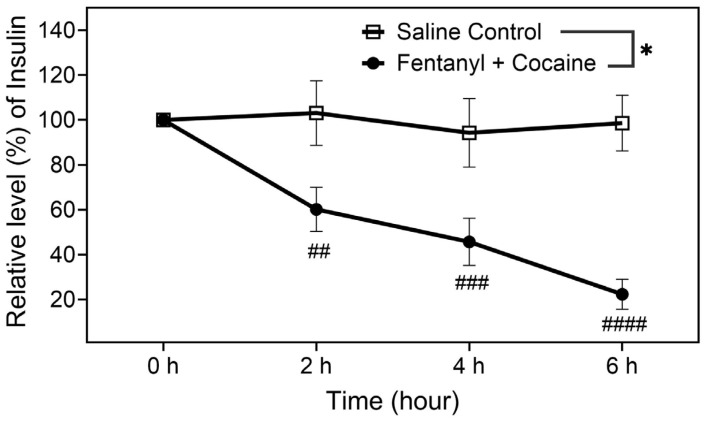
Blood levels of insulin in catheterized rats after receiving 25 manual infusions of the fentanyl–cocaine mixture at a unit dose of 2.5 µg/kg/infusion of fentanyl and 0.32 mg/kg/infusion of cocaine (n = 6) or saline (n = 9) per 2 h via IV catheters. The measured average concentrations of insulin at 0 h (pre-dose baseline) were 3.7 and 69.6 mg/dL, respectively. Statistical significance: one-way ANOVA with Dunnett post hoc analysis for comparison of the levels at various time points with the reference level at 0 h—# *p* < 0.05, ## *p* < 0.01, ### *p* < 0.001, #### *p* < 0.0001; paired *t*-test for the overall difference between the two groups of rats—* *p* < 0.05.

**Figure 4:**
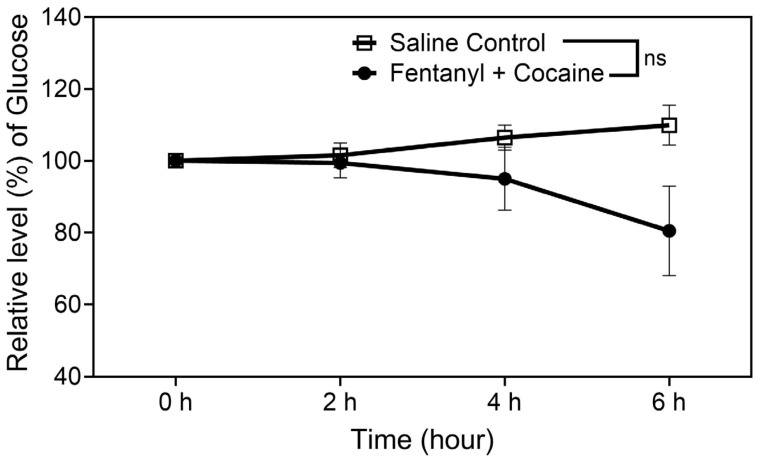
Blood levels of glucose in catheterized rats after receiving 25 manual infusions of the fentanyl–cocaine mixture at a unit dose of 2.5 µg/kg/infusion of fentanyl and 0.32 mg/kg/infusion of cocaine (n = 6) or saline (n = 9) per 2 h via IV catheters. The measured average concentrations of glucose at 0 h (pre-dose baseline) were 3.7 and 69.6 mg/dL, respectively. Statistical significance: one-way ANOVA with Dunnett post hoc analysis for comparison of the levels at various time points with the reference level at 0 h—# *p* < 0.05; paired *t*-test for the overall difference between the two groups of rats—* *p* < 0.05 (ns means that the difference is not significant with *p* > 0.05).

Further, according to the insulin data shown in Figure 3, the polydrug (mixture of fentanyl and cocaine) significantly decreased the serum insulin level. Under the influence of the polydrug, compared to the pre-dose baseline insulin level (100% as the reference) at 0 h, the remaining insulin level was only ~60% at 2 h, ~46% at 4 h, and ~22% at 6 h, as shown in Figure 3. According to the glucose data shown in Figure 4, there were no significant differences in the measured glucose levels between the polydrug and control groups. Concerning why the glucose levels did not increase while the insulin level decreased, rats did not have access to food and water during the entire experimental session within six hours, as noted above. The non-significant decreases at 4 h and 6 h time points could be associated with the observed symptoms of opioid polydrug intoxication as well.

**Figure 5:**
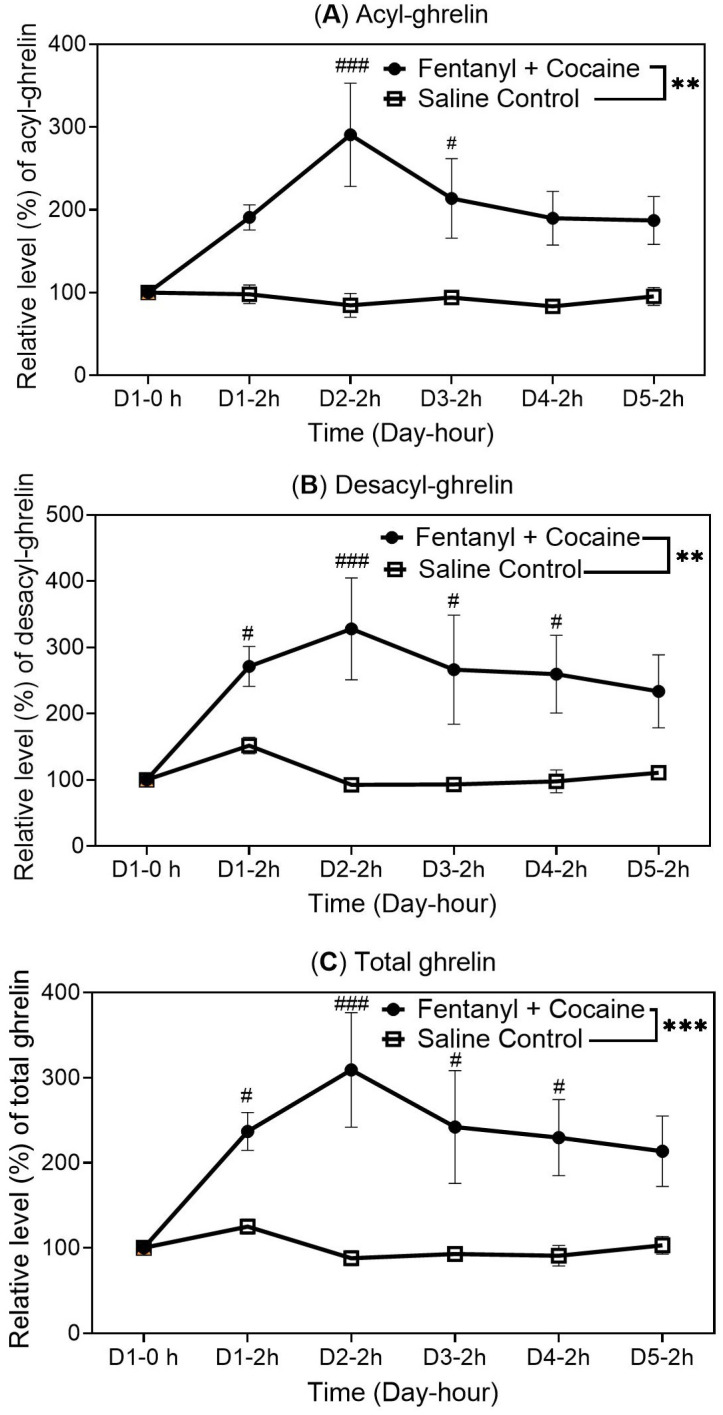
Blood (plasma) concentrations of acyl-ghrelin (**A**), desacyl-ghrelin (**B**), and total ghrelin (**C**) in catheterized rats (n = 6 per group) before saline infusion on Day 1 at 0 h (pre-dose control) and at 2 h right after receiving 25 manual infusions of the fentanyl–cocaine mixture at a unit dose of 2.5 µg/kg/infusion of fentanyl and 0.32 mg/kg/infusion of cocaine during 2 h via IV catheters for each day (Days 1 to 5). The measured average plasma concentrations of acyl-ghrelin, desacyl-ghrelin, and total ghrelin at 0 h of Day 1 (pre-dose baseline) were 218, 248, and 466 pg/mL, respectively. Statistical significance: one-way ANOVA with Dunnett post hoc analysis for comparison of the levels at various time points with the reference level at 0 h—# *p* < 0.05, ## *p* < 0.01, ### *p* < 0.001; paired *t*-test for the overall difference between the two groups of rats—* *p* < 0.05, ** *p* < 0.01, *** *p* < 0.001.

To further examine the polydrug effects on the ghrelin system, two additional groups of catheterized rats (n = 6 per group) were used to examine the polydrug effects on the ghrelin levels after multiple two-hour sessions (one session per day for five days), each session with 25 infusions of the combined 2.5 µg/kg/infusion of fentanyl and 0.32 mg/kg/infusion of cocaine for the test group and 25 infusions of saline for the control group. There were no more infusions after the 2 h time point to ensure that no rats were overdosed. The rats had no access to food or water during the entire session of two hours. For each group, the experiment started at 9 a.m. in the mornings for all the sessions. Blood (plasma) samples were collected at the 2 h time point (the end of each session) for each day (along with pre-dose blood collections at the 0 time point on Day 1 for their ghrelin baseline controls) and analyzed for their acyl-ghrelin and desacyl-ghrelin concentrations. As shown in Figure 5, along with the repeated sessions of polydrug infusions, the blood (plasma) levels of acyl-ghrelin (Figure 5A), desacyl-ghrelin (Figure 5B), and total ghrelin (Figure 5C) were all elevated significantly.

### 2.3. Ghrelin Levels in Rats Self-Administering the Polydrug of Combined Fentanyl and Cocaine

**Figure 6:**
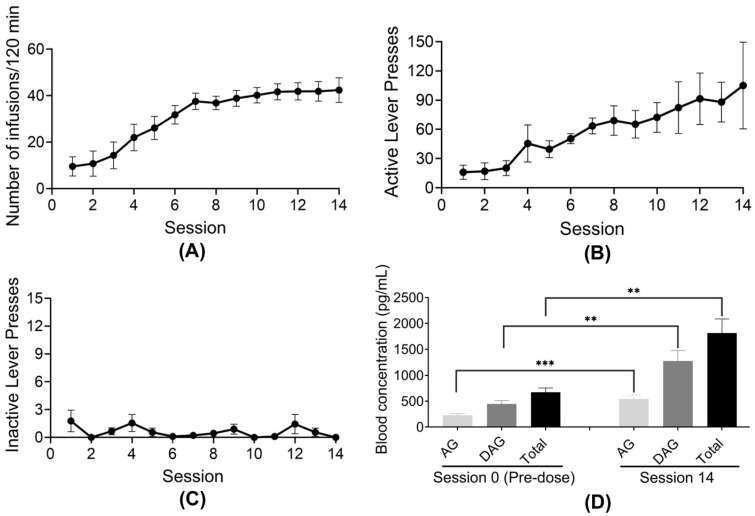
Responses of rats (n = 8) self-administering the polydrug (fentanyl–cocaine mixture with a unit dose of 1.25 µg/kg/infusion of fentanyl and 0.32 mg/kg/infusion of cocaine) under the FR1TO5 schedule in the two-hour sessions. (**A**) Number of infusions received. (**B**) Number of active lever presses. (**C**) Number of inactive lever presses. (**D**) Measured concentrations of acyl-ghrelin (AG), desacyl-ghrelin (DAG), and total ghrelin (AG + DAG = Total) on Session 14 in comparison with the pre-dose baselines on Session 0. Statistical significance (paired *t*-test between two groups): * *p* < 0.05, ** *p* < 0.01, and *** *p* < 0.001.

Eight rats (n = 8) with IV catheters were trained to self-administer the fentanyl–cocaine mixture with a unit dose of 1.25 µg/kg/infusion of fentanyl and 0.32 mg/kg/infusion of cocaine under the FR1 schedule with a time out (TO) of 5 s (FR1TO5) in daily 2-h sessions from Session 1 to Session 14, beginning at 9 a.m. for each day. Blood (plasma) samples were collected at the final session on Session 14 and analyzed for their ghrelin concentrations. To determine baseline ghrelin concentrations, on Session 0 (before the rats were trained for any drug self-administration), the rats were manually given 39 infusions of saline (based on the data in Figure 1B showing the average number of infusions associated with the unit dose of 1.25 µg/kg/infusion of fentanyl and 0.32 mg/kg/infusion of cocaine under the FR1 schedule) via the IV catheters within two hours, followed by blood (plasma) collections for the ghrelin analyses. The obtained behavioral and ghrelin data are shown in Figure 6. As shown in Figure 6, the rats quickly learned to press the active lever to receive the polydrug infusions and gradually increased the number of infusions during the daily sessions (Figure 6A–C). As seen in Figure 6D, the acyl-ghrelin, desacyl-ghrelin, and total ghrelin concentrations all significantly increased from the baseline on Session 0. From Session 0 baseline to Session 14, the average acyl-ghrelin concentration increased from 229 pg/mL to 541 pg/mL (a ~136% increase), the average desacyl-ghrelin concentration increased from 445 pg/mL to 1275 pg/mL (a ~187% increase), and the average total ghrelin concentration increased from 674 pg/mL to 1815 pg/mL (a ~169% increase).

## 3. Discussion

In this study, multiple experiments were performed to assess the effects of the fentanyl and cocaine co-use on the circulating levels of acyl-ghrelin, desacyl-ghrelin, insulin, and glucose in rats. All experimental data consistently revealed that the concurrent use of fentanyl and cocaine can significantly elevate both the acyl-ghrelin and desacyl-ghrelin levels and significantly downregulate the insulin level without significant effects on the glucose level. As pointed out by Leggio et al. [24], “upregulation of the ghrelin system seems to enhance craving for drugs as well as substance use”. Hence, the elevated ghrelin level may also enhance the rewarding effects from the combined drugs of fentanyl and cocaine in the body. Like cocaine itself, the fentanyl–cocaine polydrug can self-promote its rewarding effects via elevating the ghrelin level. For this reason, the ghrelin system might serve as a potential pharmacological target for treatment of SUDs caused by polysubstance use involving fentanyl and cocaine.

Notably, in the experiment for manually administering 75 infusions of the fentanyl–cocaine mixture corresponding to a unit dose of 2.5 µg/kg/infusion of fentanyl and 0.32 mg/kg/infusion of cocaine for 6 h (25 infusions per 2 h), the non-significant elevation of acyl-ghrelin level was observed at 2 h and was suppressed at 4 h and 6 h time points while the symptoms of opioid polydrug intoxication, such as sedation, itching, and body wounds, were observed. Concerning why the acyl-ghrelin level was suppressed at 4 h and 6 h time points, there are at least two possibilities. First, the accumulated doses of the opioid polydrug at 4 h and 6 h time points were too high (Table 1) such that the opioid polydrug intoxication suppressed the acyl-ghrelin level. On the other hand, it is also possible that the repeated administration of the polydrug might not elevate the acyl-ghrelin level. To address this question, another experiment was carried out for manually administering 25 infusions of the fentanyl–cocaine mixture corresponding to a unit dose of 2.5 µg/kg/infusion of fentanyl and 0.32 mg/kg/infusion of cocaine for only two hours per day, but the manual infusions were repeated for five days (Figure 5), demonstrating that the polydrug can always elevate the acyl-ghrelin level when the polydrug is used repeatedly. It is likely that the unexpected acyl-ghrelin levels observed at the 4 h and 6 h time points were due to the opioid polydrug intoxication. As an opioid drug, respiratory depression is the common observation in fentanyl overdose situations [25]. Intravenous administration of fentanyl alone can cause rigidity of the chest muscles which interrupts normal breathing [26]. This could slow down blood circulation and limit the ghrelin produced in the stomach being acylated by GOAT and transported into the blood system, which may explain the phenomenon that the potential elevation of the drug-induced acyl-ghrelin level was suppressed by the drug intoxication in the overdose situation. Further studies will be required to test this speculation. In a further experiment with rats self-administering the polydrug using a lower fentanyl unit dose (1.25 µg/kg/infusion instead of 2.5 µg/kg/infusion), rats were allowed to take whatever number of polydrug infusions they wanted to take, ensuring that there were no further polydrug infusions when they no longer wanted further infusions. The self-administration experiment further confirmed that the acyl-ghrelin, desacyl-ghrelin, and total ghrelin levels were also elevated by the polydrug self-administration.

Concerning the main differences between the manual administration and self-administration, each rat in each group may choose to take the polydrug (fentanyl–cocaine) infusions as many as the rat wants during a self-administration session. In comparison, by manually administering the polydrug, all rats in each group passively received a specific quantity of the combined drugs. The two different approaches consistently resulted in the increases in both acyl-ghrelin and deacyl-ghrelin levels in plasma. For this reason, possible therapeutic development for the treatment could target ghrelin by a protein drug such as an exogenous ghrelin degradation enzyme or a small-molecule drug interacting with the receptors/transporters in the ghrelin signaling network, such as a selective antagonist of ghrelin receptor GHS-R1A that seems to be upregulated along with the circulating ghrelin upregulation based on previous reports [22].

Finally, as is well known, the ghrelin level may influence insulin levels in the body. Based on the insulin data obtained in this study, the insulin level was always decreased significantly and considerably no matter whether the rats were overdosed or not (Figure 3) with the high accumulated doses. As reported in the literature, “Low Plasma Ghrelin Is Associated With Insulin Resistance, Hypertension, and the Prevalence of Type 2 Diabetes” [27]. Hence, it is expected that the fentanyl and cocaine polydrug could potentially induce cardiovascular problems, particularly for the users with insulin resistance and diabetes. Further studies will be required to examine this perspective. The combined use of fentanyl and cocaine seems to have less impact on the glucose level, which could be caused by lack of food access.

## 4. Materials and Methods

### 4.1. Animals and Drugs

Male Sprague–Dawley rats (250–270 g) were ordered from Envigo (Indianapolis, IN, USA) and allowed ad libitum access to food and water except during testing time. All rats were maintained on a 12 h light/12 h dark cycle. All experiments were performed during the light phase of the light/dark cycle in accordance with the animal protocol approved by the IACUC (Institutional Animal Care and Use Committee) at the University of Kentucky. Self-administration sessions or human manual polydrug administering started around 9 a.m. Cocaine and fentanyl hydrochloride were provided by the National Institute on Drug Abuse (NIDA) Drug Supply Program (Bethesda, MD, USA) and dissolved in physiologic saline as salt form, respectively. Acylated ghrelin (acyl-ghrelin) ELISA kits (#10006307) and unacylated ghrelin (desacyl-ghrelin) ELISA kits (#10008953) were ordered from Cayman Chemical Company, Ann Arbor, MI, USA. For ghrelin sampling, a potassium phosphate buffer (0.1 M pH 7.4 in which 1.2% NaOH (*v*/*v*) and 10 mM p-hydroxymercuribenzoic acid were added) and a 15 mg/mL EDTA solution (pH 8.0) were prepared. Ultra-sensitive rat insulin ELISA kits (#90062) were ordered from Crystal Chem, Elk Grove Village, IL, USA. FreeStyle^®^ Lite Blood Glucose Monitoring Meter and FreeStyle Lite blood glucose test stripes were used to measure blood glucose levels.

### 4.2. Apparatus

Intravenous self-administration (IVSA) and unearned intravenous repeated injection (manually administered infusions) were carried out in the same procedure room using the same set of operant conditioning chambers (29.5 cm L × 24.8 cm W × 18.7 cm H; Med Associates, St Albans, VT, USA) described in our previous paper [28]. Briefly, each self-administration chamber equipped with two retractable steel response levers was connected to the software MED-PC (Med Associates) to program experiments and collect data. Each chamber was coupled with a food pellet (#F0021; Bio-Serv, Flemington, NJ, USA) dispenser and cooperated with a variable rate syringe pump (#PHM-100VS; Med Associates) to deliver drug solution via polyurethane tubing (0.025 × 0.055 in; Instech Laboratories Inc., Plymouth Meeting, PA, USA) connected to a liquid swivel and spring tether set (#VAH95T; Instech Laboratories Inc.), which was held in place by a counterbalanced arm.

### 4.3. Intravenous Catheterization Surgery

Dustless diet pellet (#F0021; Bio-serv) was used to pre-train the rats to obtain lever–press behavior before surgery (10 sessions). Upon the surgery, rats weigh about 300 g. Intravenous catheterization surgery followed the similar procedures reported in our previous publication [28]. Intravenous catheters (#C30PU-RFV1418; Instech Laboratories, Inc.) were inserted in the left femoral vein of the rats aseptically under isoflurane anesthesia. The internal end of the catheter was secured in position by being anchored with nonabsorbable surgical sutures (# 51-7623 Size 3-0; Harvard Apparatus, Holliston, MA, USA); the distal end of the catheter exited on the back and was linked to a support harness (#VAH95AB; Instech Laboratories, Inc.) wearing around the scapula. To maintain sterility and protect the catheter, the base of the catheter was sealed with a small plastic cover cap. After surgery, analgesic Carprofen (5 mg/kg, SC) from Norbrook and antibiotic Cefazoline (20 mg/kg, IV) from WG Critical Care were injected for 3 and 7 days, respectively, and the rats were housed individually. Heparinized saline (10 U/mL, Heparin Sodium Injection, Sagent Pharmaceuticals, Schaumburg, IL, USA) was used to flush the catheters daily. Rats fully recovered from IVSA surgery within 7 days. In this study, rats had 10–14 days of recovery from surgery before SA training or manual administration of the combined drugs of fentanyl and cocaine.

### 4.4. Self-Administration Training and Dose–Response Curve

Rats with implanted IV catheters were trained to self-administer a drug solution (cocaine alone, fentanyl alone, or the cocaine and fentanyl mixture) under a schedule of fixed ratio-1 (FR1) and time out 5 s (TO-5) in daily 2-h sessions. To receive one dose of a training drug solution, rats needed to press one active (right) lever; responding on the left lever was recorded but had no programmed consequence (inactive). A 5.0 mL syringe (SKU:301027; BD Medical Systems Luer-Lok Syringe) containing a drug solution (cocaine alone or fentanyl alone or the cocaine and fentanyl mixture) was positioned tightly on the pump, which was set at RPM 5 with a flow rate of 0.7356 mL/min. After acquiring stable responding in the 120-min session, a stable baseline was achieved (i.e., with a variation of less than three infusions over the last three sessions) before further behavioral experiments. The dose–response curve of fentanyl IVSA with fixed dose of 0.32 mg/kg/inf. cocaine was obtained by conducting 2-h IVSA testing sessions with a various of fentanyl dose levels (0, 0.05, 0.5, 1.25, 2.5, and 5 µg/kg/inf.) with fixed unit dose of cocaine (0.32 mg/kg/inf.). At least three training sessions would be performed intermittently to maintain stable IVSA behavior.

### 4.5. Acute Effects of the Combined Fentanyl and Cocaine on Ghrelin, Insulin, and Glucose Levels

After full recovery from catheterization surgery, two new groups of rats (n = 9 for the saline group and n = 6 for the drug group) were used to test the acute effects of combined cocaine and fentanyl on acyl-ghrelin and desacyl-ghrelin levels in rats. The rats used in this experiment did not need to be conditioned to press the lever. The combined drug solution was injected manually and repeatedly into rats through an IV catheter by using the “operation” function of the pumps to deliver the combined drug solution over the 6-h session (75 infusions in 6 h, with 25 infusions per 2 h). The infusion number was consistent with the average infusion number that rat earned by self-administration (25 infusions in a 2-h session). The unit dose used in this experiment was 0.32 mg/kg/inf. of cocaine and 2.5 µg/kg/inf. of fentanyl. Plasma samples for ghrelin analysis and serum samples for insulin analysis were collected at 0, 2 h, 4 h, and 6 h from the saphenous vein according to the previous sampling method [29]. Ghrelin samples were collected by mixing 74 µL of blood with 20 µL of potassium phosphate buffer solution with EDTA (10 µL of potassium phosphate buffer mixed with 10 µL of EDTA solution). Ghrelin blood samples were centrifuged at 3500 rpm under 4 °C for 15 min, and then supernatants were aliquoted into two separate tubes: one for acyl-ghrelin ELISA analysis and another for desacyl-ghrelin ELISA analysis. Insulin samples were collected in clean polypropylene tubes; following centrifugation, the resulting supernatants were immediately transferred into clean polypropylene tubes. All samples should be quickly assayed or stored at −20 °C for later analysis within 6 months after collection. Samples were kept on ice between collection and centrifugation. Blood glucose level was directly measured during blood collection.

### 4.6. Effects of Daily Administering the Combined Fentanyl and Cocaine on Ghrelin Levels

After full recovery from the catheterization surgery, two new groups of rats (n = 6 for the saline group and n = 6 for the drug group) were used to test the effects of combined cocaine and fentanyl on acyl- and desacyl-ghrelin levels in rats over 6 days. A similar study design and drug solution were used in this experiment as in the last experiment. The combined drug solution was injected repeatedly into rats through an intravenous catheter by using the “operation” function of the pumps to deliver the combined drug solution over the 2-h session/day (25 infusions in 2 h) for 5 days/sessions (Days 1 to 5). The infusion number was consistent with the average infusion number that rat earned by self-administration. As in the previous experiment, the combined drug doses were 0.32 mg/kg/inf. of cocaine and 2.5 µg/kg/inf. of fentanyl. Plasma samples for ghrelin analysis and serum samples for insulin analysis were collected at 0 and 2 h on Day 0, and then at 2 h on Days 1 to 5 from saphenous vein as noted above. Blood glucose level was directly measured during blood collection. All samples were collected and handled in the same way as the above experiment.

### 4.7. Ghrelin Levels in Rats Intravenously Self-Administering the Combined Cocaine and Fentanyl

After full recovery from the catheterization surgery, a new group of rats (n = 8) was used to study the acyl- and desacyl-ghrelin levels in rats intravenously self-administering the combined cocaine and fentanyl, particularly comparing ghrelin levels on Session 0 (pre-dose) to Session 14. Rats were manually administered 39 infusions of saline in the first session. Then, rats were trained to self-administer a combined drug solution (cocaine and fentanyl) under the FR1TO5 schedule with a unit dose of 0.32 mg/kg/inf. of cocaine and 1.25 µg/kg/inf. of fentanyl for 14 sessions. Each session lasted for 2 h every day. Blood samples were collected on the saline day and the 14th session day to analyze ghrelin levels.

### 4.8. Sample Analysis

Acyl- and desacyl-ghrelin samples were analyzed following the manufacturer’s instructions using corresponding ELISA kits (96 well plates). Briefly, 12 µL of the ghrelin supernatant sample was diluted at 1:10 with EIA buffer to avoid matrix effect. Moreover, 100 µL of the diluents were dispensed to wells and mixed with 100 µL of ghrelin tracer solution. After 3 h of incubation, the plates were washed, and optimal development was obtained by adding 200 µL of Ellman’s reagent to each well. The absorbance was measured by reading the plate at a wavelength of 405 nm (yellow color) and applied to standard curve which was established following the same procedures to calculate the concentration of ghrelin in diluted unknown samples. Insulin samples were also analyzed following the manufacturer’s instructions using corresponding ELISA kits (96-well plate) based on a sandwich enzyme immunoassay protocol. Furthermore, 10 µL of serum sample was mixed with 90 µL of sample diluent after incubation for 2 h at 4 °C, and 100 µL of conjugate solution was added to the washed wells. After adding 100 µL of substrate solution and incubating for 40 min at RT, 100 µL of stop solution was added, and the color was measured at 450/620 nm. The absorbance value (450 nm subtracted 620 nm) was applied to standard curve which was established following the same procedures to calculate the concentration of insulin in unknown samples.

### 4.9. Statistical Analysis

All data were analyzed by using the GraphPad Prism 10 software (GraphPad Software, La Jolla, CA, USA). The data contained within figures are expressed as means with SEMs. The ghrelin, insulin, or glucose levels at various time points (with more than two time points in a time course) were analyzed by one-way analysis of variance (ANOVA) with Dunnett post hoc analysis. The paired *t*-test (with each time point for a pair) was used to analyze the difference between two groups of data. A difference was considered significant when *p* < 0.05 (* or # *p* < 0.05; ** or ## *p* < 0.01; *** or ### *p* < 0.001; and **** or #### *p* < 0.0001).

## Figures and Tables

**Table 1:** Accumulated doses of fentanyl and cocaine at various time points for the manual infusions via IV catheters.

Time Point	Number of IV Infusions Received	Accumulated Doses of Fentanyl and Cocaine	
		Fentanyl (mg/kg)	Cocaine (mg/kg)
0 (pre-dose)	0	0	0
2 h	25	0.063	8
4 h	50	0.125	16
6 h	75	0.188	24

## Data Availability

The datasets used and/or analyzed during the current study are available from the corresponding authors on reasonable request.
